# Systematic literature review of the signs and symptoms of respiratory syncytial virus

**DOI:** 10.1111/irv.13100

**Published:** 2023-02-05

**Authors:** Ann Colosia, Jessica Costello, Kelly McQuarrie, Kelly Kato, Kristi Bertzos

**Affiliations:** ^1^ RTI Health Solutions Research Triangle Park North Carolina USA; ^2^ RTI Health Solutions Manchester UK; ^3^ Janssen Global Services Horsham Pennsylvania USA; ^4^ Janssen Global Services Raritan New Jersey USA; ^5^ Present address: Merck & Company Rahway New Jersey USA

**Keywords:** adults, pediatrics, respiratory infection, respiratory syncytial virus, signs, symptoms

## Abstract

Respiratory syncytial virus (RSV) is responsible for over 30 million lower respiratory tract infections (LRTIs) and 3 million hospitalizations worldwide each year. Despite the risk RSV poses to young children, older adults, and individuals with comorbidities or suppressed immunity, there is limited understanding of RSV symptom presentation across these at‐risk groups, and there is no vaccine for RSV. We conducted two systematic literature reviews (SLRs) of studies that document signs and symptoms (S&S) of RSV in (1) children aged ≤5 years and (2) immunocompromised adolescents and adults, and adults at high risk for severe RSV due to age or comorbidities. Symptom duration and hospital length of stay (LOS) were explored. Electronic database searches were performed following PRISMA guidelines. Studies captured RSV S&S across community and hospital settings. Clinicians and caregivers reported (*n* = 25 studies) nasal discharge/congestion, cough, shortness of breath, feeding abnormalities, and fever in ≥40% of children across studies and settings. Median hospital stays for children ranged from 2 days in the United States to 7.5 days in China. High‐risk adults with RSV (*n* = 6 studies) commonly (≥40% of adults) reported cough, sputum, dyspnea, and fever/feverishness. Median length of hospital stay in adults ranged from 6 to 15 days across studies. Caregivers and clinicians reported similar RSV S&S in young children, including upper and lower respiratory and systemic symptoms. In high‐risk and immunocompromised adults, the most frequent (in multiple publications) and commonly reported RSV S&S were primarily LRTI symptoms. RSV symptoms could last for weeks and are variable based on geography.

## 1 INTRODUCTION

Respiratory syncytial virus (RSV) is a common respiratory illness responsible for over 30 million lower respiratory tract infections (LRTIs) and 3 million hospitalizations worldwide each year.^1^ Recently (following easing of Covid‐19 restrictions), RSV detections, emergency department visits, and hospitalizations are on the rise in the United States (US) and at least 20 countries and geographic areas have experienced intensified RSV activity.^2^, ^3^ Respiratory syncytial virus is particularly detrimental to people with compromised immune systems, chronic lung or heart disease, and people under the age of 5 years or over the age of 65 years. Respiratory syncytial virus is the leading global cause of LRTIs in children aged under 5 years and is a global leading cause of death for children under 1 year of age. In adults, RSV may cause serious infections of the lower respiratory tract and is associated with over 177 000 hospitalizations and over 14 000 deaths in the US alone every year.^4^ Evidence suggests that RSV hospitalizations are on the rise among adults in the US, particularly those aged over 65 years.^5^ Respiratory syncytial virus is known to exacerbate some serious health conditions, including asthma, chronic obstructive pulmonary disease (COPD), congestive heart failure, arrythmia, and myocardial infarction and is one of the most common causes of mortality among hematopoietic stem cell transplant recipients.^6^, ^7^ In children who contract RSV in their first 3 years of life, the risk of developing asthma is nearly three times greater than in children who do not.^8^ All of these long‐term effects related to RSV lead to significant socioeconomic burden and detriment to health‐related quality of life.^9^

Given the health risks and mortality associated with RSV, there has been decades‐long demand for effective therapeutics to treat or prevent RSV infections and related illness. In 1998, the US Food and Drug Administration approved ribavirin, an antiviral agent, but ribavirin has minimal clinical benefit in RSV and is not routinely prescribed.^10^, ^11^ The monoclonal antibody palivizumab has been approved by the US Food and Drug Administration for prevention of severe RSV in certain high‐risk pediatric patients, but none of the indications for this drug are for children over 2 years of age or for adults.^12^, ^13^ Most patients with active infections receive supportive care only. Currently, there is a great deal of ongoing research, including clinical trials, aimed at treating or preventing RSV.^13^ While there are promising products in development,^14^, ^15^, ^16^ there are currently no vaccines approved to reduce the risk of RSV, leaving only effective hygiene techniques as a prevention for most persons at this time.

Because of this lack of effective treatments and preventions, and the vulnerable populations RSV affects, a clear understanding of how RSV presents is important for identifying individuals most at risk for disease progression, identifying treatment targets, and evaluating treatment efficacy in each of these populations. To provide a comprehensive examination of the signs and symptoms (S&S) of RSV in the most at‐risk populations, two systematic literature reviews (SLRs) of patient‐reported S&S were conducted, one in young children and one in high‐risk adults (older adults or those with comorbidities) and immunocompromised adults and adolescents. Because young children are not able to report their symptoms, the literature review for this population focused on studies that included reports by clinicians and caregivers of children with RSV. This study can provide researchers and clinicians with the tools necessary to identify and adequately treat RSV infections based on the unique needs of each of these vulnerable populations.

## 2 METHODS

We performed two separate systematic reviews of literature published in English‐language journals indexed in the MEDLINE, Embase, PsychINFO, and Cochrane Library medical databases: One review of the literature from 22 June 2011 to 22 June 2021 on caregiver‐ or clinician‐reported RSV S&S in children aged ≤5 years, and one review of the literature from 21 June 2011 to 21 June 2021 on self‐reported RSV S&S in adults at high risk for RSV‐related disease progression (i.e., adults aged ≥65 years; adults with congestive heart failure, COPD, or asthma) and adults or adolescents who are immunocompromised. Additional searches of conference abstracts indexed in Embase and bibliographies of SLRs identified in the literature were performed. Included studies were limited to qualitative, focus group, and real‐world studies; clinical trials were included if they assessed patient‐reported S&S and if they included caregiver‐reported, questionnaire, measurement, or assessment terms in the title, abstract, or Medical Subject Headings (MeSH) or Emtree indexing. All sources were identified, screened, and included in this review on the basis of Preferred Reporting Items for Systematic Reviews and Meta‐Analyses (PRISMA) guidelines,^17^ as shown in Figures S1 and S2. Screening took place in two stages against the inclusion and exclusion criteria laid out in Table 1. Data on study design and characteristics, patient demographics and clinical characteristics, and outcomes of interest were then extracted from the relevant publications. Outcomes of interest included caregiver‐ and clinician‐reported RSV S&S in young children and patient‐reported RSV S&S in high‐risk adults and immunocompromised adults and adolescents in both outpatient/community and hospitalized settings. Signs and symptoms were considered common if they were reported in 40% or more of the study population. Symptom duration and hospital length of stay (LOS) for the populations of interest were extracted from articles collected in our systemic review of S&S as an exploratory objective; the literature was not searched systematically for these outcomes.

**TABLE 1:** Review of literature on pediatric and adult populations with RSV: Inclusion and exclusion criteria.

Criterion	Included	Excluded
**Pediatric population**		
Population	Studies that include pediatric patients (from newborn to 5 years of age) with RSV Studies that combine pediatric RSV populations and other populations but report data separately for pediatric patients with RSV	Studies that recruit or include only patients with a different disease or condition from that of RSV Studies of patients older than 5 years of age Studies that include adult and pediatric patients but that do not report data separately for pediatric patients aged ≤ 5 years
Interventions and comparators	None; not specific to a particular treatment	None
Outcomes	Clinical signs/symptoms	Outcomes not related to symptomatic impacts on patients
Study design	Qualitative studies Focus group studies Reviews of qualitative research^a^ Prospective and retrospective real‐world studies Randomized and nonrandomized clinical trials in which caregiver‐reported or clinician‐reported outcomes also are assessed	Randomized and nonrandomized clinical trials not assessing caregiver‐reported or clinician‐reported outcomes Nonrandomized controlled clinical trials Cross‐sectional studies Case–control studies Preclinical studies Animal studies (not in humans) Case reports Commentaries, letters, or editorials (publication type)
Language	English language	Non‐English language
Date	Published articles: past 10 years (January 2011 to June 2021) Conference abstracts: past 3 years (June 2018 to June 2021)	Articles published prior to January 2011 Conference abstracts from conferences held prior to June 2018
**Adult and adolescent populations**		
Population	Studies that include High‐risk adults (i.e., ≥65 years of age,* or ≥18 years of age with congestive heart failure, COPD, or asthma) or Immunocompromised adults or adolescents with RSV Studies that combine these populations and other populations but report data separately for adults and immunocompromised adolescents with RSV	Studies that focus on another population that also includes the populations of interest with RSV but whose results are not separated by population Studies that recruit or include only patients with a different disease or condition from that of RSV Studies of only children aged < 13 years Studies that include adult, adolescent, and pediatric patients but that do not report data separately for adults or adolescents
Interventions and comparators	None; not specific to a particular treatment	None
Outcomes	Clinical signs/symptoms	Outcomes not related to symptomatic RSV
Study design	Qualitative studies Focus group studies Reviews of qualitative research^b^ Prospective and retrospective real‐world studies Randomized and nonrandomized clinical trials in which patient‐reported outcomes also are assessed	Randomized and nonrandomized clinical trials not assessing patient‐reported outcomes Cross‐sectional studies Case–control studies Preclinical studies Animal studies (not in humans) Case reports Commentaries, letters, or editorials (publication type)
Language	English language	Non‐English language
Date	Published articles: past 10 years (21 June 2011 to 21 June 2022) Conference abstracts: past 3 years (June 2018 to June 2021)	Articles published prior to the past 10 years Conference abstracts from older conferences (those held more than 3 years ago, i.e., before 2019)

Abbreviations: COPD, chronic obstructive pulmonary disease; RSV, respiratory syncytial virus.

^a^ Reviews of qualitative research were included at level 1 screening, used for identification of primary studies, and then excluded at level 2 screening.

^b^ Upon review of the articles, studies with adults aged ≥60 years were included in the systematic literature review.

## 3 RESULTS

### 3.1 Pediatric population

#### 3.1.1 Eligible studies

We identified 651 unique publications on pediatric RSV infections through our literature search. After two levels of manual screening following the PRISMA guidelines and our inclusion and exclusion criteria (see Table 1), we found 33 publications eligible for inclusion in the pediatric review (Table S1). Eligible studies were identified across North America, western Europe, Asia, and the Middle East, as well as parts of Africa. Twenty‐five studies reported on RSV S&S in the target pediatric population. In addition, 13 studies reported symptom duration,^18^, ^19^, ^20^, ^21^, ^22^, ^23^, ^24^, ^25^, ^26^, ^27^, ^28^, ^29^, ^30^ including nine studies that also presented RSV S&S. Hospital LOS was extracted from 13 of the included studies in this review.^19^, ^21^, ^25^, ^26^, ^30^, ^31^, ^32^, ^33^, ^34^, ^35^, ^36^, ^37^, ^38^

#### 3.1.2 S&S in all pediatric populations

Studies on S&S of RSV in the pediatric population (25 studies) took place in both community and clinical settings, and S&S were reported by both caregivers (six studies)^19^, ^20^, ^21^, ^39^, ^40^, ^41^ and clinicians (22 studies)^19^, ^20^, ^21^, ^26^, ^27^, ^28^, ^29^, ^34^, ^35^, ^37^, ^38^, ^39^, ^40^, ^42^, ^43^, ^44^, ^45^, ^46^, ^47^, ^48^, ^49^, ^50^; we analyzed and present these two sets separately. In addition, two publications presented clinician‐reported S&S in the intensive care unit (ICU).^24^, ^51^ The studies we identified varied considerably in the types of S&S evaluated, age group, treatment setting, and geographical location. Nasal discharge, nasal congestion, cough, shortness of breath, feeding abnormalities, and fever were the most commonly reported S&S by both clinicians and caregivers, occurring in at least 40% of children across multiple studies and treatment settings (Tables S1 to S3).^19^, ^21^, ^28^, ^29^, ^31^, ^34^, ^35^, ^39^, ^40^, ^41^, ^42^, ^43^, ^46^, ^47^, ^49^, ^50^, ^52^

#### 3.1.3 Caregiver‐reported S&S in the community setting

**FIGURE 1:**
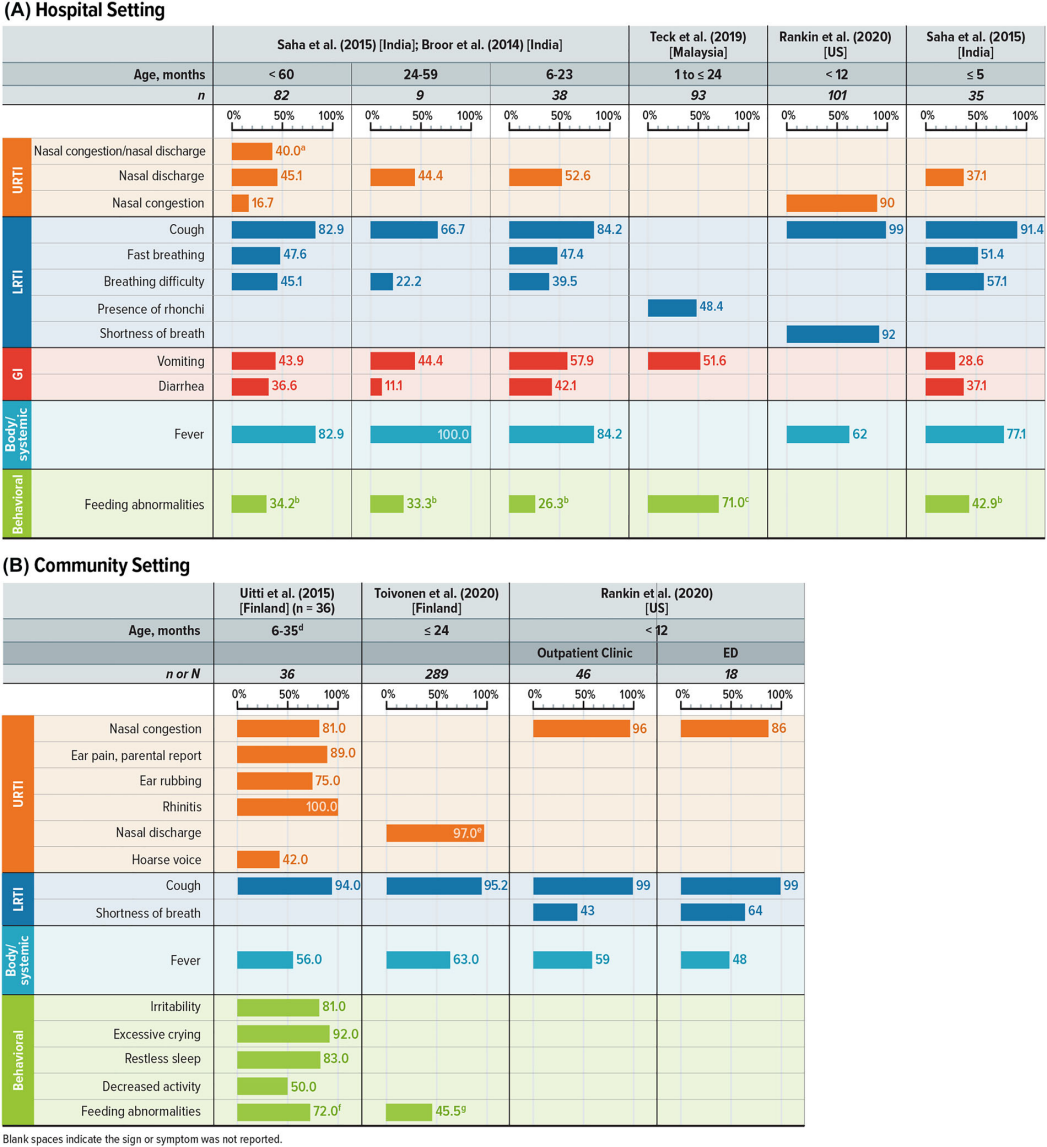
Caregiver‐reported RSV S&S in young children. LRTI = lower respiratory tract infection; RSV = respiratory syncytial virus; S&S = signs and symptoms; URTI = upper respiratory tract infection; US = United States. Notes: Figure shows the percentage of patients reporting the listed sign or symptom in each study. Symptoms were included if at least one study reported any in at least 40% of children. Blank spaces indicate the sign or symptom was not reported. ^a^Reported in Broor et al^32^ (India) when the number of RSV+ children was 50. ^b^Reported as inability/refusal to eat. ^c^Reported as poor feeding. ^d^Patients were included in this study if their parents suspected acute otitis media. ^e^Reported as rhinorrhea. ^f^Reported as poor feeding. ^g^Reported as poor appetite.

Four studies presented S&S reported by caregivers of children with RSV initially and primarily treated in the outpatient setting (Table S2).^20^, ^21^, ^39^, ^41^ All four studies were prospective and enrollment ranged from 361 to 923 children. One of these studies reported solely on wheezing.^20^ In the other three studies evaluating a range of RSV symptoms (Figure 1), cough was reported in all of the studies and in 94% to 99% of children.^21^, ^39^, ^41^ Nasal congestion or discharge and fever were reported across all three of these studies as well.^21^, ^39^, ^41^ Feeding abnormalities, reported as poor appetite, were reported by caregivers in two studies, occurring in 72% of children aged 6 to 35 months with signs of acute otitis media^41^ and 45.5% of children aged ≤24 months.^21^ Although only reported by caregivers in one study, shortness of breath was reported in infants more frequently in the emergency department (64%) than in outpatient clinics (43%).^39^

#### 3.1.4 Caregiver‐reported S&S in the hospital setting

Four publications representing three studies presented S&S reported by caregivers of children hospitalized with RSV (Figure 1 and Table S2)^19^, ^31^, ^39^, ^40^; two publications^31^, ^40^ reported on overlapping study populations. All three studies were prospective and ranged in enrollment from 245 to 412 children. No symptom was identified across all three studies, partly because one study, Teck et al^19^ (Malaysia), reported only three symptoms: presence of rhonchi, vomiting, and feeding abnormalities (reported as poor feeding). However, several symptoms were reported by caregivers in two of the three studies, including cough (over 66.7% of children), fever (over 62% of children), feeding abnormalities (over 26% of children), and vomiting (over 28% of children).

Saha et al^40^ presented caregiver‐reported symptoms for children in different age categories, with nine children in the 24–59 month age category, 38 patients in the 6–23 months age category, and 35 patients in the ≤5 months age category. Caregivers noted fever at higher rates in the older age categories: 77.1% of children aged 0 to 5 months, 84.2% of those aged 6 to 23 months, and 100% of the children aged 24 to 59 months. The percentage of children reported by caregivers as having a cough associated with RSV decreased as children got older: 91.4% of those aged 0 to 5 months, 84.2% of those aged 6 to 23 months, and 66.7% of the children aged 24 to 59 months. Reports of breathing difficulty decreased as age increased: 57.1% of children aged 0 to 5 months, 39.5% of those aged 6 to 23 months, and 22.2% of the children aged 24 to 59 months.

#### 3.1.5 Clinician‐reported S&S in the community setting

**FIGURE 2:**
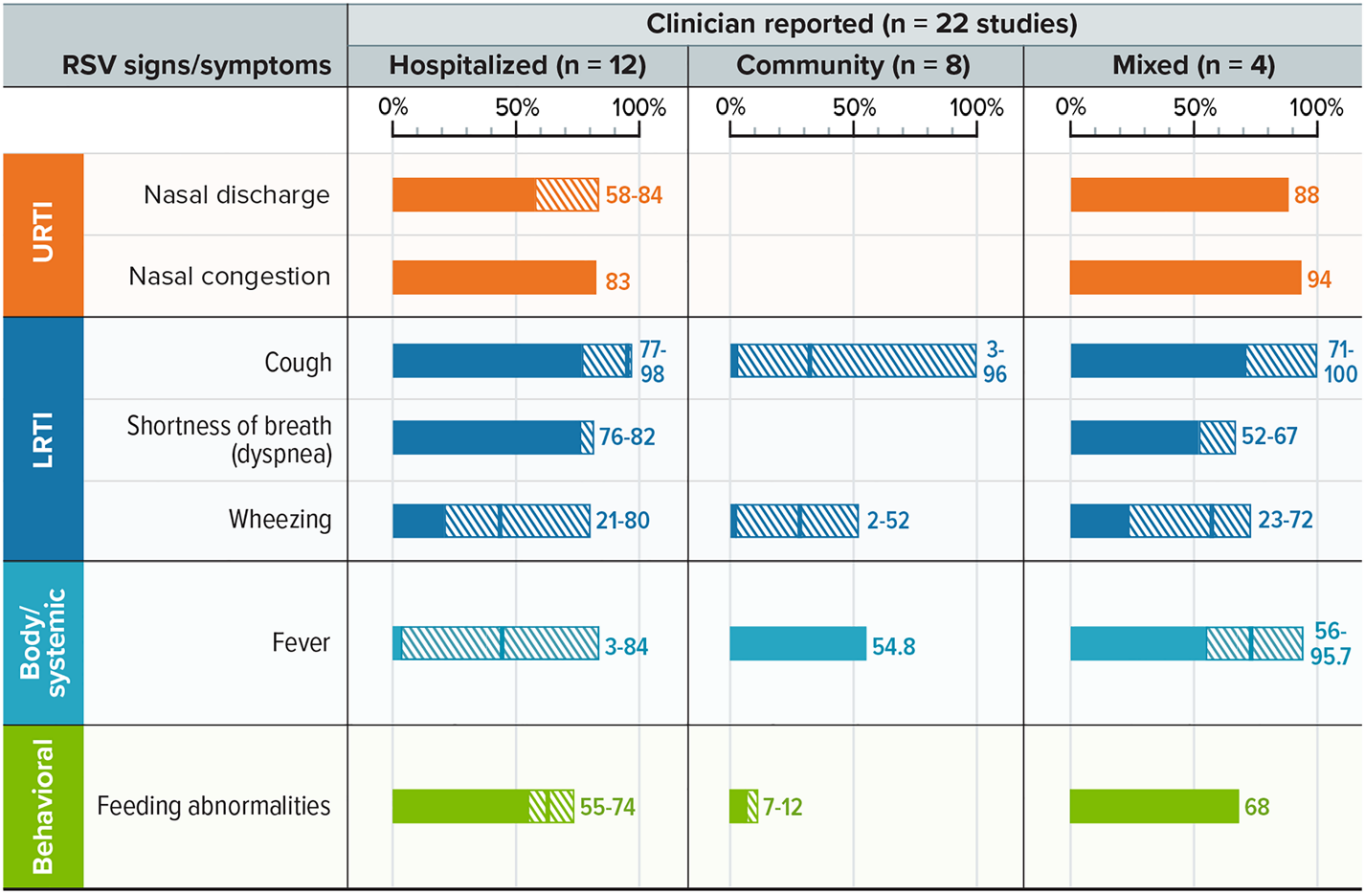
Clinician‐reported RSV S&S in young children. LRTI = lower respiratory tract infection; RSV = respiratory syncytial virus; S&S = signs and symptoms; URTI = upper respiratory tract infection. Notes: Excluding two studies in the intensive care unit setting. For each sign/symptom, the figure shows the range of the percentage of patients reporting the symptom across studies. Symptoms were included if at least one study reported any in at least 40% of children. Blank spaces indicate the sign or symptom was not reported.

In the non‐hospitalized setting, eight studies captured clinician‐reported RSV S&S in children, primarily in infants.^20^, ^21^, ^26^, ^27^, ^39^, ^45^, ^49^, ^50^ Table S3 shows RSV S&S commonly reported by clinicians in the community or mixed settings. The RSV‐positive (RSV+) populations in these studies ranged in size from 11 to 311. Figure 2 shows the most common S&S across age groups, which include nasal discharge/congestion, cough, fever, and feeding abnormalities. In infants, cough (96%) and wheezing (52%) were common in one study,^49^ but cough was uncommon (3%–4%) in another study,^27^ and wheezing was less common in three other studies (2.2%–36.6%).^20^, ^21^, ^26^, ^39^, ^45^ No study included in this review specified RSV S&S particular to outpatient children aged 3 to 5 years, but in a single study in Kenya with children under 5 years of age, fever and crackles were found in over 40% of children.^50^

#### 3.1.6 Clinician‐reported S&S in the hospital setting

In the hospital setting (not including patients in the ICU^24^, ^51^), there were 12 studies of clinician‐reported RSV S&S in children aged 0 to 5 years (Figure 2 and Table S4).^19^, ^26^, ^28^, ^34^, ^35^, ^37^, ^39^, ^40^, ^43^, ^44^, ^46^, ^48^ Studies in the ICU setting are summarized separately below. The most common S&S (≥40% of patients) reported by clinicians in the hospitalized pediatric population were cough, nasal discharge or congestion, dyspnea, and feeding abnormalities.^28^, ^34^, ^35^, ^37^, ^43^, ^46^, ^48^

Fever was also a commonly identified symptom of RSV by clinicians in the hospitalized pediatric population,^34^, ^35^, ^37^, ^40^, ^44^, ^46^ although, similar to caregiver reports, it appeared to follow an age‐dependent trend, increasing with age. Conversely, wheezing was reported for a higher percentage of children aged 0 to 5 months compared with those aged 6 to 23 months and 24 to 59 months, a finding indicating a strong age dependence^40^ and supported by other studies.^26^, ^29^, ^39^, ^40^, ^48^ One US study^39^ also found that wheezing, the one symptom identified in all studies of children aged less than 1 year,^26^, ^39^, ^40^, ^48^ was reported more frequently in the hospitalized population than the outpatient population (74% vs. 28%; *P* < 0.001).^24^, ^26^, ^39^, ^40^, ^48^, ^51^ Saha et al^40^ also found that fever was more common as the age category increased (2.9% in those aged 0–5 months; 26.3% in those aged 6–23 months; 66.7% in those aged 24–59 months).

Other trends were also identified based on clinician reports in the hospital setting. Tachypnea^31^, ^34^, ^40^ was most frequently reported in children aged 0 to 5 months (71.0%), less frequently reported in children aged 6 to 23 months (42.1%), and least frequently reported in children aged 24 to 59 months (11.1%).^40^ The overall rate of tachypnea for hospitalized children studied was 51.2%.^34^, ^40^ Hypoxia was found to be more prevalent in severe RSV infections and ranged from 20.7% of children with non‐severe infections to 64.7% of children with severe infections.^35^

In 2 studies, clinicians reported RSV S&S for children in the ICU.^24^, ^51^ One prospective single‐center study in a neonatal ICU in Turkey found that nasal congestion, vomiting, nasal discharge, and cyanosis were reported in over 40% of infants.^24^ An Israeli study reported that a cyanotic event was present in 36.2% to 41.4% of infants in the intensive care setting.^51^

#### 3.1.7 Clinician‐reported S&S in mixed settings

Four studies captured clinician reports of pediatric RSV S&S in a mixed setting (hospitalized and non‐hospitalized), including children aged 0 to 4 years.^28^, ^38^, ^42^, ^47^ The sample sizes ranged from 23 to 152 children. In at least two studies, cough, shortness of breath, wheezing, and fever were all reported in over 40% of children (Table S3).^28^, ^38^, ^42^, ^47^

#### 3.1.8 Length of hospital stay and symptom duration

Hospital LOS was reported in 13 publications; three reported mean and median LOS,^26^, ^30^, ^34^ four reported only mean,^19^, ^33^, ^35^, ^36^ and six reported only median.^21^, ^25^, ^31^, ^32^, ^37^, ^38^ Both mean and median hospital stay were approximately 4 to 5 days for most countries, but the LOS was longer in studies based in Malaysia and China, where hospital stays were 7.27 days (mean) and 7.5 days (median), respectively.^19^, ^30^

Some studies suggested factors potentially impacting LOS in pediatric populations. In a study conducted in the US,^35^ when mechanical ventilation was required, the mean LOS was 12.7 days, as compared with 2.6 days for those who did not require assisted ventilation. In another study conducted in Malaysia, Ng et al^34^ identified symptoms that were associated with differences in LOS. Children who presented with fever or rhinorrhea had a shorter mean hospital LOS (4.5 days each), while children under 5 years presenting with tachypnea had a longer mean hospital LOS (5.2 days).^34^

Thirteen studies reported RSV symptom duration,^18^, ^19^, ^20^, ^21^, ^22^, ^23^, ^24^, ^25^, ^26^, ^27^, ^28^, ^29^, ^30^ with 11 studies reporting durations of any symptoms (i.e., duration of illness).^18^, ^19^, ^20^, ^21^, ^22^, ^25^, ^26^, ^27^, ^28^, ^29^, ^30^ However, the methods used to capture symptom duration varied among the studies, as did the time periods during which symptom duration was reported, making comparisons difficult. For example, for clinician‐reported symptom duration, some studies reported symptom duration prior to seeking treatment, but did not track symptom duration for the entire disease course.^26^, ^29^ When reported, the median overall duration of any symptoms reported in studies in the community setting included in this SLR ranged from 4 to 12 days (Table S5).

### 3.2 Adult population

#### 3.2.1 Eligible studies

Our search identified 234 unique studies of RSV infections in high‐risk or immunocompromised adults. Through the PRISMA‐guided screening process outlined in Figure S2, eight studies were included in this literature review (Table S1).^47^, ^53^, ^54^, ^55^, ^56^, ^57^, ^58^, ^59^ Eligible studies spanned North America, Europe, and East Asia. Results are presented for high‐risk adults and immunocompromised populations separately. For this SLR, patients were considered at “high risk” for RSV‐related disease progression if they were of advanced age (≥60 or ≥65 years, depending on the study) or had specific comorbidities (e.g., cardiovascular disease, lung disease, asthma, COPD, and diabetes). Of the eight studies, six reported the percentage of patients with various RSV S&S in high‐risk populations; however, data were not presented by risk factor (i.e., age or comorbidity). One study was conducted in the community setting,^53^ two in the hospital setting,^54^, ^55^ and three in a mix of both settings.^56^, ^57^, ^60^ Two additional studies measured RSV S&S in immunocompromised patients.^58^, ^61^ One of these studies included both adolescents and adults (aged 15 and over), although the mean age of the sample was 49 years, suggesting a limited sample of adolescents, and S&S were not presented separately by age group.^58^ Therefore, RSV S&S in immunocompromised adolescents cannot be reported. The second study evaluating RSV S&S in immunocompromised adults reported RSV symptoms patients experienced prior to receiving treatment (oral or inhaled ribavirin) but did not present the proportions of patients with these different symptoms.^58^

#### 3.2.2 S&S in all high‐risk adult populations

**FIGURE 3:**
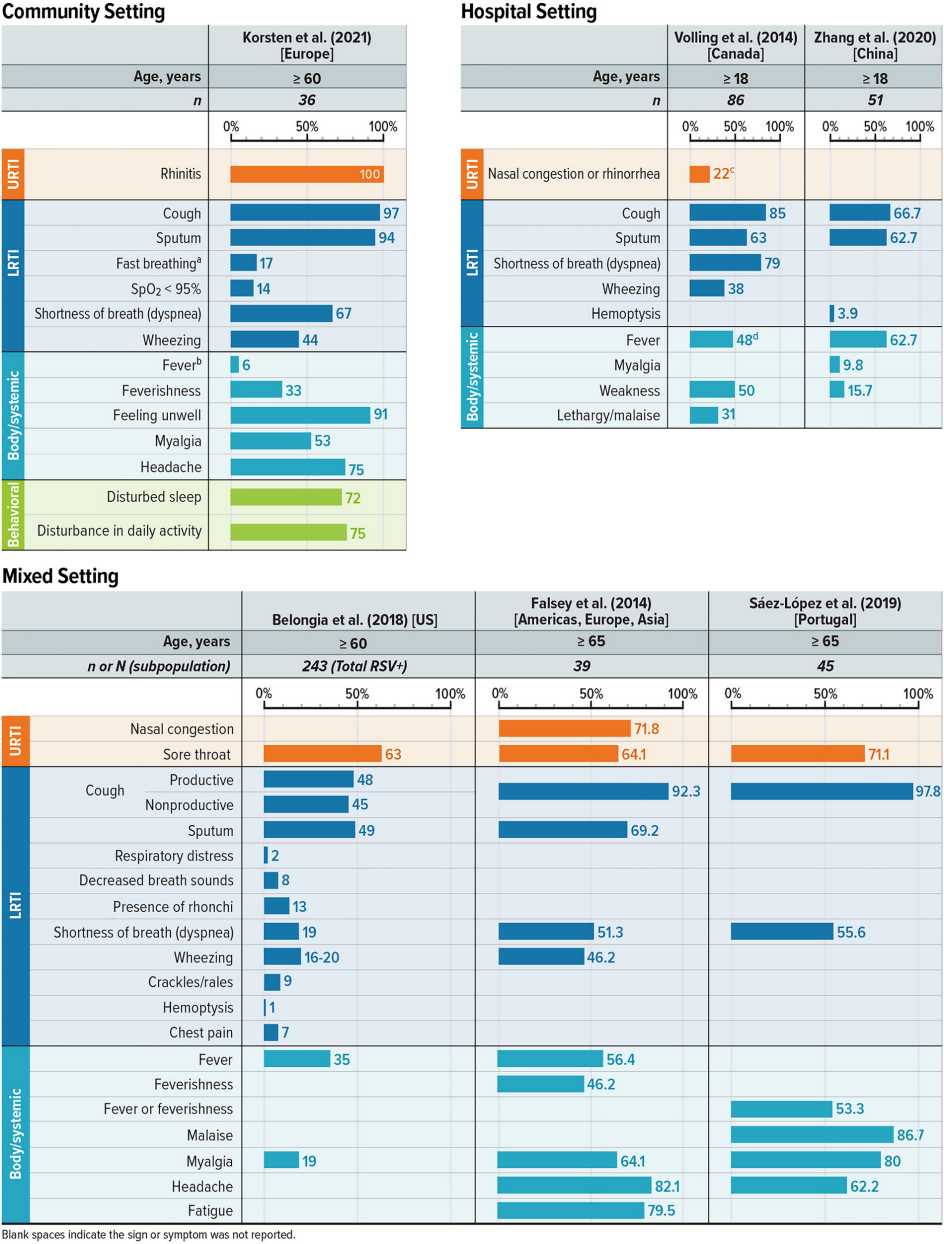
Patient‐reported RSV S&S in high‐risk adults. LRTI = lower respiratory tract infection; RSV = respiratory syncytial virus; S&S = signs and symptoms; URTI = upper respiratory tract infection; US = United States. Note: Figure shows the percentage of patients reporting the listed sign or symptom in each study. Symptoms were included if at least one study reported it in at least 40% of children. Blank spaces indicate the sign or symptom was not reported. ^a^Reported as respiratory rate >20 breaths·min^−1^. ^b^Reported as fever ≥38°C. ^c^Reported as runny nose or nasal congestion. ^d^Reported as fever (≥38.0°C) in emergency department.

Figure 3 shows the range of RSV S&S self‐reported by adults at high risk for RSV‐related disease progression. The majority of reported symptoms across all treatment settings (hospital, community, and mixed) were LRTI symptoms; proportions of LRTI symptoms were similar in both community and hospital settings (Figure 3). The only symptom consistently reported across all settings and in all studies was cough, with 66.7% to 97.8% of high‐risk adults with RSV reporting this symptom. Sputum was also commonly reported by 49% to 94% of adults in five of the six studies reviewed.

#### 3.2.3 S&S in high‐risk adults in the community setting

Only one study presented S&S reported by patients with RSV treated in the community setting, which focused on European countries and included 1040 adults aged ≥60 years.^53^ Rhinitis was the only upper respiratory tract infection (URTI) symptom presented in the study, and it occurred in all patients. Cough was the most common LRTI symptom, occurring in 97% of patients; conversely, fast breathing (defined as respiratory rate >20 breaths per minute) (17%) and oxygen saturation (SpO_2_) < 95% (14%) were reported the least. Feeling unwell was reported in 91% of high‐risk adults, and disturbances in sleep and daily activities were reported in 72% and 75% of patients, respectively.^53^

#### 3.2.4 S&S in high‐risk adults in the hospital setting

Two studies presented S&S reported by patients with RSV treated in the hospital setting; one was conducted in Canada^54^ and the other in China.^55^ Both were retrospective cohort studies that reviewed electronic and paper medical records to obtain the data for the study. The following symptoms were reported in both studies: cough, sputum, fever, and weakness (Figure 3). In addition, Volling et al^54^ reported dyspnea (79%), wheezing (38%), lethargy/malaise (31%), and nasal congestion or rhinorrhea (22%), and Zhang et al^55^ reported myalgia (9.8%) and hemoptysis (3.9%).

#### 3.2.5 S&S in high‐risk adults in mixed settings

Three studies included patients in both hospital and outpatient settings.^56^, ^57^, ^60^ Belongia et al^56^ (US) and Falsey et al^57^ (global) were prospective studies, whereas the Saez‐Lopez et al^60^ (Portugal) study was an observational retrospective study of the Portuguese influenza surveillance system. This system included primary healthcare centers, general hospitals with pediatric and adult emergency departments and medical wards, and a pediatric hospital. However, the proportions of patients in a hospitalized versus community setting included in this analysis were not provided. The study by Saez‐Lopez et al^47^ included 45 adults aged ≥65 years with RSV, the study by Falsey et al^57^ included 556 adults aged ≥65 years with moderate‐to‐severe influenza‐like illness, and the study by Belongia et al^56^ included 241 adults aged ≥60 years with RSV.

Two LRTI symptoms (cough [92.3%–97.8%] and shortness of breath [19%–55.6%]) were reported in all three studies.^56^, ^57^, ^60^ Sputum (49%–69.2%) and wheezing (16%–46.2%) were reported in two of the studies.^56^, ^57^ Additional LRTI symptoms were presented only in the Belongia study and ranged from 1% (hemoptysis) to 13% (presence of rhonchi).^56^ The only URTI symptom reported by patients in all three studies was sore throat, ranging from 63% to 71.1% of patients.^56^, ^57^, ^60^ Nasal congestion was reported in 71.8% of patients in one study.^57^ Common body/systemic symptoms included fever (with or without feverishness) and myalgia, which were reported in all three studies at rates of 35%–56.4% and 19%–80%, respectively.^56^, ^57^, ^60^ Headache was reported in two studies (62.2%–82.1%).^57^, ^60^ Malaise and fatigue were each reported in one study in 86.7% and 79.5% of patients, respectively.^57^, ^60^

#### 3.2.6 S&S in immunocompromised patients

Only two studies presented S&S in exclusively immunocompromised populations^58^, ^61^; in one study, all but two patients were hospitalized,^58^ and another study took place in a mixed setting.^61^ While one of these studies of immunocompromised patients (after lung transplant) included adolescents aged >15 years, the mean age of the sample was 49,^58^ so no inferences can be made about the RSV S&S experienced by immunocompromised adolescents.

Of the two studies, one was a retrospective analysis of adult lung transplant patients infected with RSV receiving oral or inhaled ribavirin. This study did not report proportions of patients experiencing each of the RSV S&S, but did report fever, cough, dyspnea, wheezing, rhinorrhea, pharyngitis, and headache as present in both treatment groups.^58^ The second study, Marcelin et al,^61^ was the only study to report the proportions of immunocompromised patients with each RSV symptom; cough was reported in 94% of patients, fever was seen in 62% of patients, and dyspnea was seen in 59%.

#### 3.2.7 Length of hospital stay and symptom duration

In addition to reporting S&S of RSV, six of the identified studies examined the hospital LOS and/or duration of RSV symptoms in high‐risk adults with RSV. Regarding hospital LOS, Belongia et al^56^ reported a mean hospital stay of 3.5 days (standard deviation [SD], 2.5 days) in the US. Two studies recorded the median hospital LOS. In the Falsey et al^57^ study conducted globally, the median LOS for older adults with RSV was 6 days (range, 3–20 days), although breakdown by country was not provided. In the retrospective cohort study conducted in China by Zhang et al,^55^ median LOS was reported as 15 days (interquartile range, 13–22). A Canadian retrospective cohort study by Volling et al^54^ presented both mean and median values for LOS: 10.8 days (SD, 16.7) and 6 days (range, 1–140), respectively. Duration of symptoms relating to RSV in high‐risk adults was only reported in two studies.^53^, ^59^ Symptom duration varied from 17 to 19 days. The studies were conducted globally, so the results suggest that symptom duration does not vary substantially by geography.

In immunocompromised patients with RSV undergoing lung transplants, mean hospital LOS was 11 days (SD, 15.1) in the six patients receiving oral ribavirin and 5 days (SD, 1.5) in the 15 patients receiving inhaled ribavirin.^58^ No evidence regarding the duration of symptoms or symptom burden in immunocompromised patients was reported.

## 4 DISCUSSION

This comprehensive SLR captured S&S of RSV infection in pediatric populations, across a variety of ages and reported by both caregivers and clinicians, and high‐risk or immunocompromised adult populations. Across all reviewed ages (pediatric and adult), settings (hospital, community, or mixed), and reporters (self, caregiver, or clinician), cough was the most commonly and frequently reported RSV symptom. Studies on the impact of RSV S&S on health‐related quality of life have also identified cough as not only one of the most prevalent symptoms of RSV but also one of the most burdensome symptoms in both children and adults.^59^, ^62^, ^63^, ^64^

Among studies of caregiver‐ and clinician‐reported RSV S&S in children, regardless of setting or age group, RSV signs or symptoms that were commonly reported (≥40%) and also reported by at least three studies of each study type (caregiver‐reported or clinician‐reported) were cough, fever, and feeding abnormalities. While wheezing is also considered a prominent symptom of RSV in children,^65^ we found that it was commonly reported in children by clinicians, but not by caregivers; it is possible that caregivers may have captured wheezing as part of reporting “breathing difficulties” rather than using the term wheezing.

Symptom trends based on age or treatment setting in the pediatric population were overall not robust. The most salient trend identified in this review was related to fever. Both clinicians and caregivers more commonly reported fever in hospitalized children as their age increased.^29^, ^34^, ^35^, ^37^, ^40^, ^44^, ^46^, ^48^ Trends were difficult to evaluate in this review due to the different methodologies implemented for the various studies (e.g., differences in breakdown of age categories and RSV S&S assessed).

Overall, caregivers and clinicians reported similar S&S with similar frequency, despite the wide variety of RSV symptoms reported, indicating that caregivers may be acceptable reporters for their children, who are often unable to accurately report symptoms themselves.

The studies of adults with RSV identified by this review focused primarily on adults over the age of 60 years. Four of the seven studies identified for the adult population included patients with comorbidities such as cardiovascular disease, lung disease, asthma, COPD, and diabetes, in addition to advanced age.^53^, ^54^, ^55^, ^56^ In high‐risk adults, the most common RSV S&S identified in this review were LRTI symptoms, including cough (which was identified in all studies), sputum, and dyspnea. Other common symptoms included fever and myalgia. Although we initially included immunocompromised adolescents in our literature search of RSV S&S, only one study included immunocompromised adolescents (patients 15 years of age and older). However, the mean age of patients in the study was 49 years, so data were not available for immunocompromised adolescents in this SLR.^58^ Due to the limited number of studies identified in this review for adults, no trends in RSV S&S could be identified by risk factor or across treatment setting.

Our review found that hospital LOS varied based on geography for both hospitalized adult and pediatric patients. Hospital LOS was examined in six studies of adult patients hospitalized for RSV across the globe. Across high‐risk and immunocompromised patients, mean LOS varied from 3.5 to 11 days in the US/Americas^56^; the median LOS varied from 6 days in the US/Americas^54^, ^57^ to 15 days in China.^55^ In children, the length of hospital stay was typically 4–5 days,^25^, ^26^, ^31^, ^32^, ^33^, ^34^, ^37^, ^38^ except for those hospitalized in China and Malaysia, where LOS extended to approximately a week.^19^, ^30^ Considering the different healthcare systems and protocols across the different countries and age groups, variation was to be expected for LOS.

Reports of RSV symptom duration in children varied significantly, from 4 to 12 days, and depended on the method of evaluation, so no overall trends could be identified.^20^, ^21^, ^22^, ^27^, ^28^, ^30^ Our review found that symptoms in adults lasted several weeks (17–19 days).^53^, ^59^ However, given that symptom duration may not have been monitored following hospital discharge, it is possible that these studies did not adequately capture the length of time that hospitalized patients may experience RSV symptoms. Considering the high prevalence of LRTI symptoms found in adults in this review, and the relative lack of reporting on duration of symptoms, the prolonged evidence of symptoms following discharge may be of concern to the thousands of adults who contract RSV each year and whose quality of life may be impacted for months following infection.

A recent prospective cohort study of adults with respiratory tract infections across 12 countries that was published after our literature review was completed also found that LRTI symptoms are commonly associated with RSV infections in adults, particularly cough, wheezing, and shortness of breath.^66^ In our review study, reports of wheezing were variable; while high‐risk adults reported experiencing wheezing in four of the six studies reviewed, it was only a commonly reported symptom (reported by ≥40% of adults) in two studies. The prospective cohort study also found that patients with risk factors including cardiopulmonary comorbidities experienced higher rates of LRTI symptoms. Of note, RSV infections led to more severe LRTI symptoms in adult patients compared with influenza.^66^ Furthermore, LRTI symptoms including cough and shortness of breath were observed in high‐risk adult patients with RSV at 3 months after their hospital discharge (the last follow‐up visit).^66^

This study has some limitations. We identified common RSV S&S in multiple populations but did not capture the severity or burden of those symptoms on patients; less common S&S may be more burdensome. We limited our search to English‐language studies published between 2011 and 2021, so global studies in a non‐English language would have been missed in our search. We therefore may have overrepresented studies in English‐speaking countries. Despite the availability of studies on RSV S&S, data on the duration of symptoms or the LOS in hospitals was less readily available. Studies that did report symptom duration used different criteria for evaluating duration, so it is challenging to draw conclusions. However, duration of S&S and LOS were exploratory and were not part of the search terms for this study. An SLR focused on these outcomes may provide additional details. Although we initially included immunocompromised adolescents in our literature search strategy, the lack of data on S&S separately in this population in this SLR may have been caused by our requirement that included studies were either questionnaires or clinical trials to ensure that patients were being queried about the reported S&S. Observational studies in adults likely were also omitted. Due to the resulting limited number of studies in adult and adolescent populations, as well as the variation in the list of S&S reported, firm conclusions may not be drawn on the presentation of RSV in these populations.

## 5 CONCLUSION

Cough was reported in the majority of RSV patients across all age and risk groups assessed in this study. The most frequent commonly reported RSV S&S in the pediatric population were consistent across caregivers and clinicians and across settings (nasal discharge/congestion, cough, shortness of breath, fever, feeding abnormalities). The most frequent commonly reported RSV S&S across settings in high‐risk and immunocompromised adult patients were primarily LRTI symptoms (e.g., cough, sputum, and shortness of breath). Duration of RSV S&S in children was reported as up to 12 days in this review. In high‐risk and immunocompromised adults, RSV S&S were found to last several weeks (17–19 days in this SLR). Length of hospital stay for both children and adults varied by geography.

## AUTHOR CONTRIBUTIONS

**Ann Colosia:** Conceptualization; data curation; formal analysis; investigation; methodology; project administration; writing‐original draft; writing‐review and editing. **Jessica Costello:** Conceptualization; data curation; formal analysis; investigation; methodology; writing‐review and editing. **Kelly McQuarrie:** Investigation; writing‐review and editing. **Kelly Kato:** Conceptualization; funding acquisition; methodology; project administration; supervision; writing‐review and editing. **Kristi Bertzos:** Conceptualization; formal analysis; investigation; methodology; project administration; writing‐review and editing.

## CONFLICT OF INTEREST

AC and JC are full time employees of RTI Health Solutions, an independent nonprofit research organization, which was retained by Janssen to conduct the research, which is the subject of this manuscript. Their compensation is unconnected to the studies on which they work. KB and KK are employees of Janssen and may hold shares and/or stock options in the company. KM was an employee of Janssen at the time the research was conducted and may hold shares and/or stock options in the company.

### PEER REVIEW

The peer review history for this article is available at https://publons.com/publon/10.1111/irv.13100.

## Supporting information


**Figure S1.** Flow Diagram of Article Inclusion Following PRISMA Guidelines for the Pediatric Literature Review
PRISMA = Preferred Reporting Items for Systematic Reviews and Meta‐Analyses.
**Figure S2.** Flow Diagram of Article Inclusion Following PRISMA Guidelines for the Adult Literature Review
PRISMA = Preferred Reporting Items for Systematic Reviews and Meta‐Analyses.
**Table S1.** Summary of Eligible Studies
N/A = not applicable; S&S = signs and symptoms.
Note: studies may be counted in more than one column, so the sum of columns 4–8 does not equal the total number of studies in column 3.
^a^ The study populations in Saha et al. and Broor et al. overlapped and are counted here as 1 study across 2 publications.
**Table S2.** Common (≥ 40%) Caregiver‐Reported RSV S&S in Young Children
**✓** = reported in ≥ 40% of patients; < = reported in < 40% of patients; 0 = no cases; GI = gastrointestinal; LRTI = lower respiratory tract infection; NR = not reported; RSV = respiratory syncytial virus; S&S = signs and symptoms; URTI = upper respiratory tract infection; US = United States.
Note: Thomas et al.^20^, Finland is not represented in the table; the only caregiver‐reported RSV sign or symptom presented was expiratory wheezing at home, which occurred in 28.4% of children aged ≤ 24 months treated in the outpatient setting.
^a^ Patients were included in this study if their parents suspected acute otitis media. ^b^ Reported as rhinorrhea. ^c^ None of the studies described vomiting, so whether vomiting was related to coughing or was a separate GI issue could not be determined. ^d^ Reported as poor feeding. ^e^ Reported as poor appetite.
**Table S3.** Common (≥ 40%) Clinician‐Reported RSV S&S in Children in the Community Setting or a Mix of Settings
**✓** = reported in ≥ 40% of patients; < = reported in < 40% of patients; GI = gastrointestinal; LRTI = lower respiratory tract infection; NR = not reported; RSV = respiratory syncytial virus; S&S = signs and symptoms; URTI = upper respiratory tract infection; US = United States.
^a^ Reported as runny nose. ^b^ Reported as fever or feverishness. ^c^ Reported as documented fever. ^d^ Reported as decreased appetite.
**Table S4.** Common (≥ 40%) Clinician‐Reported S&S in RSV‐Hospitalized Children
**✓** = reported in ≥ 40% of patients; < = reported in < 40% of patients; GI = gastrointestinal; LRTI = lower respiratory tract infection; NR = not reported; RSV = respiratory syncytial virus; S&S = signs and symptoms; URTI = upper respiratory tract infection; US = United States.
^a^ Data are for RSV‐positive patients only, not co‐infected patients. ^b^ Reported as rhinorrhea. ^c^ Reported as rhinorrhea or congestion. ^d^ Reported as shortness of breath.
^e^ Reported as wheezing on auscultation. ^f^ None of the studies described vomiting, so whether vomiting was related to coughing or was a separate GI issue could not be determined. ^g^ Reported as poor feeding. ^h^ Reported as feeding difficulties before admission. ^i^ Reported as reduced dietary intake. ^j^ Reported as poor appetite.
**Table S5.** Duration of Any RSV Symptoms in the Pediatric Population
ED = emergency department; IQR = interquartile range; NR = not reported; RSV = respiratory syncytial virus; SD = standard deviation; SE = standard error; US = United States.
^a^ Belgium, Bulgaria, Chile, Colombia, Croatia, Czech Republic, Germany, Hungary, Israel, Latvia, Malaysia, Philippines, Poland, Slovakia, Spain, and Thailand.
^b^ In this study, most patients were kept in the hospital until complete recovery, although some patients were discharged before complete recovery for social reasons. Therefore, authors of this SLR assume that mean hospital length of stay (7.27 days [SD 4.458 days]) is only an approximation of the duration of symptoms during hospitalization.
^c^ All consecutive days in which the child had fever, rhinitis, or cough.
^d^ Mean duration of symptoms exceeded mean duration of hospital stay for each RSV severity group.
^e^ Timing of illness duration is not explicitly stated in the conference abstract, but the table appears to contain baseline information about the patients.
^f^ Timing not explicitly stated in this conference abstract.
^g^ Fever, cough, wheeze, difficulty breathing.

## Data Availability

Data sharing is not applicable to this article, as no new data were created or analyzed in this study.
