# Supplementary material for: Systematic literature review of the signs and symptoms of respiratory syncytial virus
Source: Influenza Other Respir Viruses. 2023 Feb 5;17(2):e13100. doi: 10.1111/irv.13100 (PMC9899685; doi:10.1111/irv.13100)
Supplement: Supplementary file 1 — Figure S1. Flow Diagram of Article Inclusion Following PRISMA Guidelines for the Pediatric Literature Review PRISMA = Preferred Reporting Items for Systematic Reviews and Meta‐Analyses. Figure S2. Flow Diagram of Article Inclusion Following PRISMA Guidelines for the Adult Literature Review PRISMA = Preferred Reporting Items for Systematic Reviews and Meta‐Analyses. Table S1. Summary of Eligible Studies N/A = not applicable; S&S = signs and symptoms. Note: studies may be counted in more than one column, so the sum of columns 4–8 does not equal the total number of studies in column 3. a The study populations in Saha et al. and Broor et al. overlapped and are counted here as 1 study across 2 publications. Table S2. Common (≥ 40%) Caregiver‐Reported RSV S&S in Young Children ✓ = reported in ≥ 40% of patients; < = reported in < 40% of patients; 0 = no cases; GI = gastrointestinal; LRTI = lower respiratory tract infection; NR = not reported; RSV = respiratory syncytial virus; S&S = signs and symptoms; URTI = upper respiratory tract infection; US = United States. Note: Thomas et al.20, Finland is not represented in the table; the only caregiver‐reported RSV sign or symptom presented was expiratory wheezing at home, which occurred in 28.4% of children aged ≤ 24 months treated in the outpatient setting. a Patients were included in this study if their parents suspected acute otitis media. b Reported as rhinorrhea. c None of the studies described vomiting, so whether vomiting was related to coughing or was a separate GI issue could not be determined. d Reported as poor feeding. e Reported as poor appetite. Table S3. Common (≥ 40%) Clinician‐Reported RSV S&S in Children in the Community Setting or a Mix of Settings ✓ = reported in ≥ 40% of patients; < = reported in < 40% of patients; GI = gastrointestinal; LRTI = lower respiratory tract infection; NR = not reported; RSV = respiratory syncytial virus; S&S = signs and symptoms; URTI = upper respiratory tract infection; US = Unite [file IRV-17-e13100-s001.docx]

# SUPPLEMENTARY MATERIAL

Figure S-1. Flow Diagram of Article Inclusion Following PRISMA Guidelines for the Pediatric Literature Review


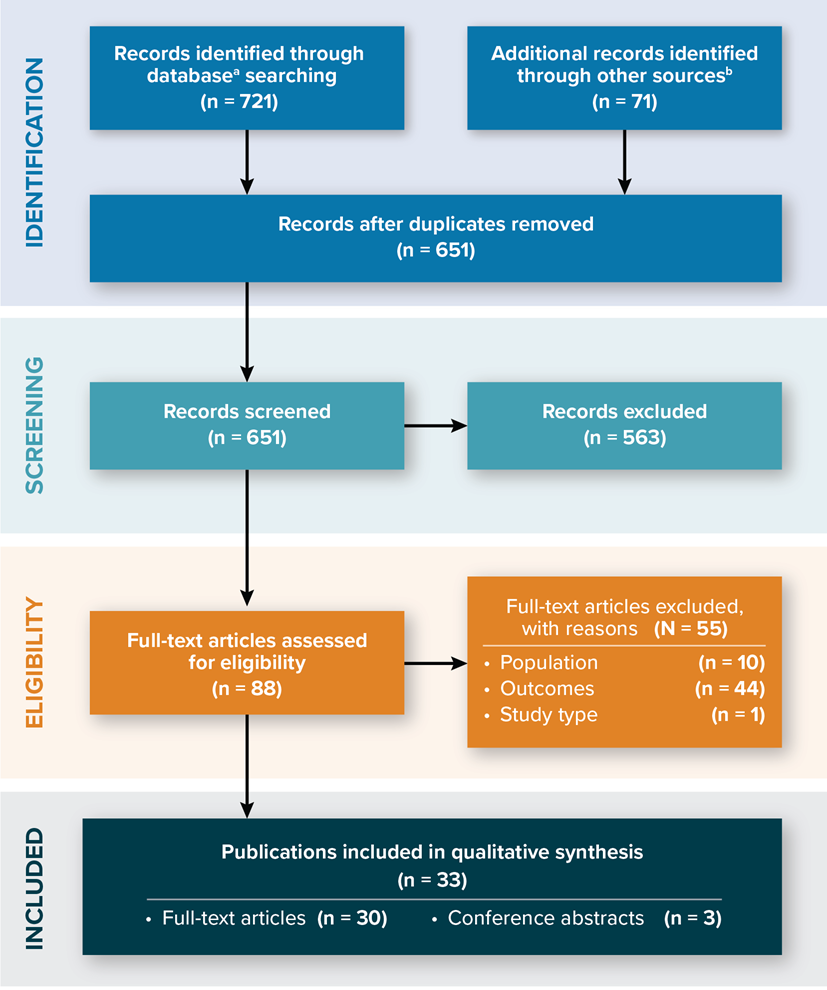


PRISMA = Preferred Reporting Items for Systematic Reviews and Meta-Analyses.

Figure S-2. Flow Diagram of Article Inclusion Following PRISMA Guidelines for the Adult Literature Review


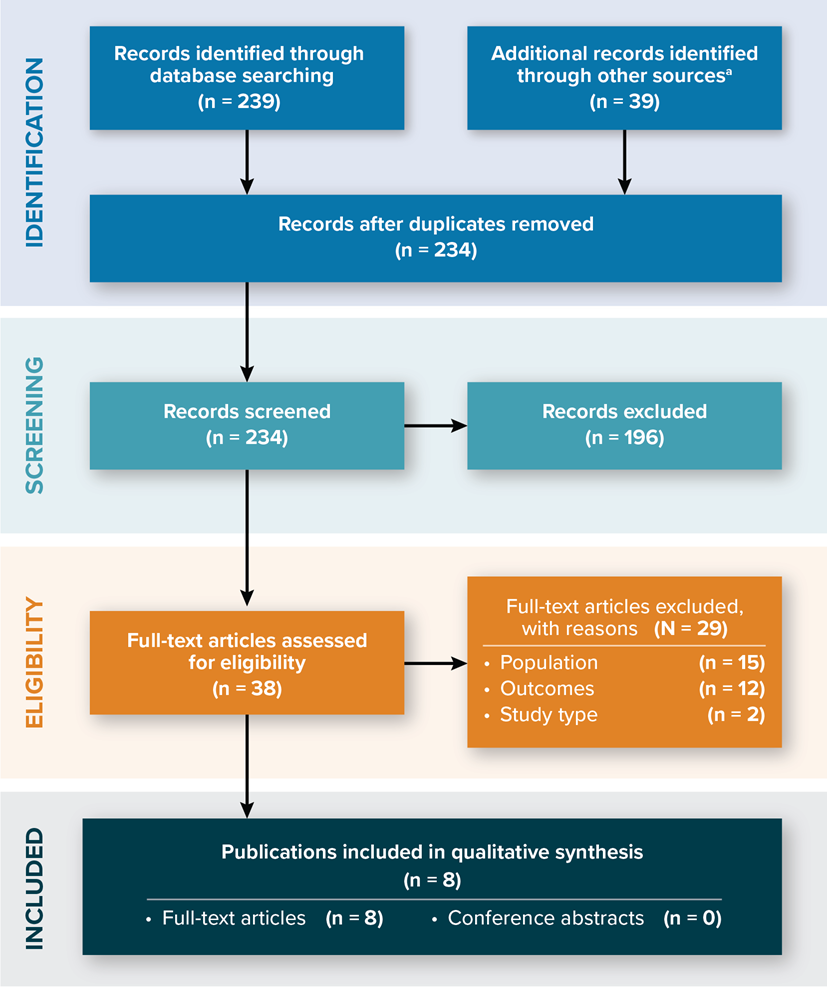


PRISMA = Preferred Reporting Items for Systematic Reviews and Meta-Analyses.

Table S-1. Summary of Eligible Studies

| **Population** | **Total eligible publications** | **No. of studies^a^** | **Studies reporting S&S** | **Studies reporting caregiver S&S** | **Studies reporting clinician S&S** | **Studies reporting length of hospital stay** | **Studies reporting symptom duration** |
| --- | --- | --- | --- | --- | --- | --- | --- |
| Pediatric | 33 | 32 | 25 | 6 | 24 | 13 | 13 |
| Adult | 8 | 8 | 8 | N/A | N/A | 5 | 2 |

N/A = not applicable; S&S = signs and symptoms.

Note: studies may be counted in more than one column, so the sum of columns 4-8 does not equal the total number of studies in column 3.

^a^ The study populations in Saha et al. and Broor et al. overlapped and are counted here as 1 study across 2 publications.

Table S-2. Common (≥ 40%) Caregiver-Reported RSV S&S in Young Children

| Sign and symptoms | | Saha S, Pandey BG, Choudekar A*et al* [1]; Broor S, Dawood FS, Pandey BG*et al* [2] India | Teck KS, Mac Guad R, Van Rostenberghe AH, Hua GS [3] Malaysia | | Rankin DA, Haddadin Z, Lipworth L*et al* [4] US | | | Toivonen L, Karppinen S, Schuez-Havupalo L*et al* [5] Finland | | Uitti JM, Tähtinen PA, Laine MK, Huovinen P, Ruuskanen O, Ruohola A [6]^a^ Finland |
| --- | --- | --- | --- | --- | --- | --- | --- | --- | --- | --- |
|  |  | Hospitalized | | | | | Community | | | |
| Age, months | | < 60 | | 1 to ≤ 24 | | < 12 | < 12 | ≤ 24 | 6-35 | |
| URTI | Nasal congestion | ✓ | | NR | | ✓ | ✓ | NR | ✓ | |
|  | Nasal discharge | ✓ | | NR | | NR | NR | ✓^b^ | NR | |
|  | Rhinitis | NR | | NR | | NR | NR | NR | ✓ | |
|  | Ear pain | NR | | NR | | NR | NR | NR | ✓ | |
|  | Ear rubbing | NR | | NR | | NR | NR | NR | ✓ | |
|  | Hoarse voice | NR | | NR | | NR | NR | NR | ✓ | |
| LRTI | Cough | ✓ | | NR | | ✓ | ✓ | ✓ | ✓ | |
|  | Shortness of breath | NR | | NR | | ✓ | ✓ | NR | NR | |
|  | Fast breathing | ✓ | | NR | | NR | NR | NR | NR | |
|  | Breathing difficulty | ✓ | | NR | | NR | NR | NR | NR | |
|  | Presence of rhonchi | NR | | ✓ | | NR | NR | NR | NR | |
| GI | Vomiting^c^ | ✓ | | ✓ | | NR | NR | < | 0 | |
| Body/ systemic | Fever | ✓ | | NR | | ✓ | ✓ | ✓ | ✓ | |
| Behavior | Feeding abnormalities | < | | ✓^d^ | | NR | NR | ✓^e^ | ✓^e^ | |
|  | Excessive crying | NR | | NR | | NR | NR | NR | ✓ | |
|  | Restless sleep | NR | | NR | | NR | NR | NR | ✓ | |
|  | Irritability | NR | | NR | | NR | NR | NR | ✓ | |
|  | Decreased activity | NR | | NR | | NR | NR | NR | ✓ | |

✓ = reported in ≥ 40% of patients; < = reported in < 40% of patients; 0 = no cases; GI = gastrointestinal; LRTI = lower respiratory tract infection; NR = not reported; RSV = respiratory syncytial virus; S&S = signs and symptoms; URTI = upper respiratory tract infection; US = United States.

Note: Thomas E, Mattila JM, Lehtinen P, Vuorinen T, Waris M, Heikkinen T [7, Finland] is not represented in the table; the only caregiver-reported RSV sign or symptom presented was expiratory wheezing at home, which occurred in 28.4% of children aged ≤ 24 months treated in the outpatient setting.

^a^ Patients were included in this study if their parents suspected acute otitis media. ^b^ Reported as rhinorrhea. ^c^ None of the studies described vomiting, so whether vomiting was related to coughing or was a separate GI issue could not be determined. ^d^ Reported as poor feeding. ^e^ Reported as poor appetite.

Table S-3. Common (≥ 40%) Clinician-Reported RSV S&S in Children in the Community Setting or a Mix of Settings

| Sign and symptoms | | Okiro EA, Ngama M, Bett A, Nokes DJ [8] Kenya | Heikkinen T, Ojala E, Waris M [9] Finland | | Toivonen L, Karppinen S, Schuez-Havupalo L*et al* [5] Finland | Heikkinen T, Ojala E, Waris M [9] Finland | | Omer SB, Bednarczyk R, Kazi M*et al* [10] Pakistan | Bashir U, Alam MM, Sadia H, Zaidi SS, Kazi BM [11] Pakistan | Sáez-López E, Pechirra P, Costa I*et al* [12] Portugal | Zar HJ, Nduru P, Stadler JAM*et al* [13] South Africa | Haddadin Z, Rankin DA, Lipworth L*et al* [14] US |
| --- | --- | --- | --- | --- | --- | --- | --- | --- | --- | --- | --- | --- |
|  |  |  | Community setting | | | | | | Mixed setting | | | |
| Age | | < 60  months | < 36  months | 24 to < 36  months | ≤ 24 months | 12-24  months | < 12  months | < 12  months | < 5  years | 0-4  years | ≤ 2  years | < 1  year |
| URTI | Acute otitis media | NR | ✓ | ✓ | < | ✓ | ✓ | NR | NR | NR | NR | NR |
|  | Nasal congestion | NR | NR | NR | NR | NR | NR | NR | NR | NR | NR | ✓ |
|  | Nasal discharge | NR | NR | NR | NR | NR | NR | NR | NR | NR | NR | ✓^a^ |
| LRTI | Cough | < | NR | NR | NR | NR | NR | ✓ | ✓ | ✓ | NR | ✓ |
|  | Crackles | ✓ | NR | NR | NR | NR | NR | NR | NR | NR | NR | NR |
|  | Crackles/rales/ rhonchi | NR | NR | NR | NR | NR | NR | NR | NR | NR | NR | ✓ |
|  | Wheezing | < | NR | NR | < | NR | NR | ✓ | ✓ | NR | ✓ | < |
|  | Difficult breathing | NR | NR | NR | NR | NR | NR | NR | ✓ | NR | NR | NR |
|  | Shortness of breath | NR | NR | NR | NR | NR | NR | NR | NR | ✓ | NR | ✓ |
|  | Lower chest indrawing | < | NR | NR | NR | NR | NR | NR | NR | NR | ✓ | NR |
| GI | GI symptoms | NR | NR | NR | NR | NR | NR | NR | NR | NR | NR | ✓ |
| Body/ systemic | Fever | ✓ | NR | NR | NR | NR | NR | NR | ✓ | ✓^b^ | NR | ✓^c^ |
| Behavior | Feeding abnormalities | NR | NR | NR | NR | NR | NR | NR | NR | NR | NR | ✓^d^ |
|  | Lethargy | NR | NR | NR | NR | NR | NR | NR | NR | NR | NR | ✓ |

✓ = reported in ≥ 40% of patients; < = reported in < 40% of patients; GI = gastrointestinal; LRTI = lower respiratory tract infection; NR = not reported; RSV = respiratory syncytial virus; S&S = signs and symptoms; URTI = upper respiratory tract infection; US = United States.

^a^ Reported as runny nose. ^b^ Reported as fever or feverishness. ^c^ Reported as documented fever. ^d^ Reported as decreased appetite.

Table S-4. Common (≥ 40%) Clinician-Reported S&S in RSV-Hospitalized Children

| Sign and symptoms | Ng KF, Tan KK, Sam ZH, Ting GS, Gan WY [15] Malaysia | Niles D, Larsen B, Balaji A*et al* [16] US | Saha S, Pandey BG, Choudekar A*et al* [1] India | Bamberger E, Srugo I, Abu Raya B*et al* [17]^a^ Israel | Pale M, Nacoto A, Tivane A*et al* [18] Mozambique | Van Leeuwen JC, Goossens LK, Hendrix RM, Van Der Palen J, Lusthusz A, Thio BJ [19] Netherlands | Faber TE, Kamps AW, Sjoerdsma MH, Vermeulen S, Veeger NJ, Bont LJ [20] Netherlands | Haddadin Z, Rankin DA, Yanis A*et al* [21] Jordan | Teck KS, Mac Guad R, Van Rostenberghe AH, Hua GS [3] Malaysia | Caserta MT, Qiu X, Tesini B*et al* [22] US | Zhang XB, Liu LJ, Qian LL*et al* [23] China | Rankin DA, Haddadin Z, Lipworth L*et al* [4] US |
| --- | --- | --- | --- | --- | --- | --- | --- | --- | --- | --- | --- | --- |
|  | Hospitalized | | | | | | | | | | | |
| Age, months | ≤ 60 | | | ≤ 24 | | | | < 24 | 1-24 | < 12 | | |
| URTI |  |  |  |  |  |  |  |  |  |  |  |  |
| Nasal discharge | ✓^b^ | ✓^c^ | NR | NR | ✓^b^ | NR | NR | ✓^b^ | NR | NR | NR | NR |
| LRTI |  |  |  |  |  |  |  |  |  |  |  |  |
| Cough | ✓ | ✓ | NR | NR | ✓ | NR | NR | ✓ | NR | NR | ✓ | NR |
| Tachypnea | ✓ | NR | ✓ | NR | NR | NR | NR | NR | NR | NR | < | NR |
| Dyspnea | NR | NR | NR | NR | ✓ | NR | NR | ✓^d^ | NR | NR | NR | NR |
| Apnea | < | NR | NR | NR | NR | NR | NR | ✓ | NR | < | < | NR |
| Crepitations | NR | NR | ✓ | NR | NR | NR | NR | NR | NR | NR | NR | NR |
| Wheezing | NR | < | ✓ | ✓^e^ | NR | NR | NR | ✓ | NR | ✓ | < | < |
| Prolonged expiration on auscultation | NR | NR | NR | ✓ | NR | NR | NR | NR | NR | NR | NR | NR |
| Crackles on auscultation | NR | NR | NR | ✓ | NR | NR | NR | NR | NR | NR | NR | NR |
| Respiratory distress | NR | NR | NR | NR | < | ✓ | NR | NR | NR | NR | NR | NR |
| Retractions | NR | NR | < | NR | NR | NR | NR | NR | NR | ✓ | NR | NR |
| Rales/rhonchi | NR | NR | NR | NR | NR | NR | NR | NR | NR | ✓ | NR | ✓ |
| Course rales | NR | NR | NR | NR | NR | NR | NR | NR | NR | NR | ✓ | NR |
| Hypoxia | NR | ✓ | < | NR | NR | NR | ✓ | NR | NR | NR | NR | NR |
| GI |  |  |  |  |  |  |  |  |  |  |  |  |
| Vomiting^f^ | NR | NR | NR | NR | NR | NR | NR | ✓ | NR | NR | NR | NR |
| Body/systemic |  |  |  |  |  |  |  |  |  |  |  |  |
| Fever | ✓ | ✓ | < | NR | ✓ | ✓ | ✓ | < | NR | NR | < | NR |
| Behavior |  |  |  |  |  |  |  |  |  |  |  |  |
| Feeding abnormalities | ✓^g^ | NR | NR | ✓^h^ | NR | ✓^i^ | NR | ✓^j^ | NR | NR | NR | NR |
| Irritability | NR | NR | NR | NR | NR | NR | NR | ✓ | NR | NR | NR | NR |
| Fatigue | NR | NR | NR | NR | NR | NR | NR | ✓ | NR | NR | NR | NR |

✓ = reported in ≥ 40% of patients; < = reported in < 40% of patients; GI = gastrointestinal; LRTI = lower respiratory tract infection; NR = not reported; RSV = respiratory syncytial virus; S&S = signs and symptoms; URTI = upper respiratory tract infection; US = United States.

^a^ Data are for RSV-positive patients only, not co-infected patients. ^b^ Reported as rhinorrhea. ^c^ Reported as rhinorrhea or congestion. ^d^ Reported as shortness of breath.

^e^ Reported as wheezing on auscultation. ^f^ None of the studies described vomiting, so whether vomiting was related to coughing or was a separate GI issue could not be determined. ^g^ Reported as poor feeding. ^h^ Reported as feeding difficulties before admission. ^i^ Reported as reduced dietary intake. ^j^ Reported as poor appetite.

Table S-5. Duration of Any RSV Symptoms in the Pediatric Population

| Reporter/Study | Setting | N | Time Assessed (Method) | Duration Measure | Days |
| --- | --- | --- | --- | --- | --- |
| ***Caregivers*** |  |  |  |  |  |
| Cunningham S, Piedra PA, Martinon-Torres F*et al* [24]  16 countries^a^ | Hospital | 175 | - Days from symptom onset to first dose of drug, which was given with 27 hours of arrival to hospital or ED (method of symptom duration determination not stated) | Mean (SD) | Ages 1-24 months   - Placebo, 3.2 (1.2) - All ALX-0171 doses, 3.2 (0.9) to 3.3 (1.2) - All children, 3.3 (1.1) |
|  |  |  | - Time back to normal (parent diaries, start time is day of first study dose; last entry is day 28 [last study visit]) | Median (IQR) | Ages 1-24 months   - Placebo, 8.4 (7.2-22.0) - All ALX-0171 doses, 9.1 (5.3-7.4) |
| Haddadin Z, Rankin DA, Yanis A*et al* [21] Jordan | Hospital | 276 | - RSV illness duration at admission^e^ (parental interviews) | Mean (SD) | Ages < 2 years, 3.6 (2.0) |
| Teck KS, Mac Guad R, Van Rostenberghe AH, Hua GS [3]^b^  Malaysia | Hospital | 93 | - Duration from start of symptoms to hospital admission (caregiver completed questionnaire) | Mean (SD) | Ages 1-24 months, 3.540 (2.060) |
| Caserta MT, Qiu X, Tesini B*et al* [22] US | Hospital and community | 84 | Duration from illness onset to first study visit, which occurred within 24 hours of hospitalization or RSV diagnosis (parent report) | Mean (SE) | - Hospitalized, ages 0.5-9.4 months, 4.7 (0.21) - Community, ages 1.1-8.9 months, 4.6 (0.28) |
| Toivonen L, Karppinen S, Schuez-Havupalo L*et al* [5]  Finland | Community | 289 | Overall duration of RSV symptoms^c^ (daily symptom diaries) | Median (IQR) | - Ages ≤ 24 months, 10.0 (8.0-14.0) - Ages 12-24 months, 11.0 (9.0-15.0) - Ages ≤ 11 months, 10.0 (8.0-13.0) |
| Thomas E, Mattila JM, Lehtinen P, Vuorinen T, Waris M, Heikkinen T [7]  Finland | Community | 134 | Overall duration of RSV symptoms (daily symptom diaries) | Mean (SD) | Ages < 12 months, 12.0 (5.7) |
| Uusitupa E, Waris M, Heikkinen T [25]  Finland | Community | 150 | Overall duration of RSV symptoms^c^ (daily symptom diaries) | Median | High viral load   - Ages 24-36 months, 10.1 - Ages < 24 months, 11.6   Low viral load   - Ages 24-36 months, 8.1 - Ages < 24 months, 11.1 |
| ***Clinicians*** |  |  | Overall duration of RSV |  |  |
| Bhuiyan MU, Luby SP, Alamgir NI*et al* [26] Bangladesh | Hospital | 39 | Days from symptom onset to hospitalization (NR) | Median (IQR) | Ages, < 5 years, 3 (2-5) |
| Zhou L, Xiao Q, Zhao Y, Huang A, Ren L, Liu E [27] China | Hospital | 40 | Duration of RSV symptoms^d^ (NR) | Mean (SD) | Ages ≤ 2 years   - Mild RSV with bronchiolitis, 11.5 (1.3) - Moderate RSV with bronchiolitis, 10.2 (1.2) - Severe RSV with bronchiolitis, 11.89 (2.5) |
| Haddadin Z, Rankin DA, Lipworth L*et al* [14]  US | Hospital or community | 101 | RSV illness duration^f^ (parent interviews and medical chart abstraction) | Mean (SD) | Ages < 1 year, 3.9 (2.2) |
| Chu HY, Katz J, Tielsch J*et al* [28]  Nepal | Community | 311 | Overall duration of RSV symptoms^g^ | Median (range) | - Ages 2-6 months, 5 (0-21) - Ages 0-2 months, 4 (0-26) |

ED = emergency department; IQR = interquartile range; NR = not reported; RSV = respiratory syncytial virus; SD = standard deviation; SE = standard error; US = United States.

^a^ Belgium, Bulgaria, Chile, Colombia, Croatia, Czech Republic, Germany, Hungary, Israel, Latvia, Malaysia, Philippines, Poland, Slovakia, Spain, and Thailand.

^b^ In this study, most patients were kept in the hospital until complete recovery, although some patients were discharged before complete recovery for social reasons. Therefore, authors of this SLR assume that mean hospital length of stay (7.27 days [SD 4.458 days]) is only an approximation of the duration of symptoms during hospitalization.

^c^ All consecutive days in which the child had fever, rhinitis, or cough.

^d^ Mean duration of symptoms exceeded mean duration of hospital stay for each RSV severity group.

^e^ Timing of illness duration is not explicitly stated in the conference abstract, but the table appears to contain baseline information about the patients.

^f^ Timing not explicitly stated in this conference abstract.

^g^ Fever, cough, wheeze, difficulty breathing.

# Supplemental References

1. Saha S, Pandey BG, Choudekar A *et al*. Evaluation of case definitions for estimation of respiratory syncytial virus associated hospitalizations among children in a rural community of northern India. *J Glob Health*. 5(2), 010419 (2015).

2. Broor S, Dawood FS, Pandey BG *et al*. Rates of respiratory virus-associated hospitalization in children aged <5 years in rural northern India. *J Infect*. 68(3), 281-289 (2014).

3. Teck KS, Mac Guad R, Van Rostenberghe AH, Hua GS. Prevalence, risk factors and clinical characteristics of respiratory syncytial virus-associated lower respiratory tract infections in Kelantan, Malaysia. *J Med Virol*. 91(9), 1608-1615 (2019).

4. Rankin DA, Haddadin Z, Lipworth L *et al*. Comparison of clinical presentations and burden of respiratory syncytial virus in infants across three distinct healthcare settings. *Open Forum Infect Dis*. 7(suppl 1), S711 (2020).

5. Toivonen L, Karppinen S, Schuez-Havupalo L *et al*. Respiratory syncytial virus infections in children 0-24 months of age in the community. *J Infect*. 80(1), 69-75 (2020).

6. Uitti JM, Tähtinen PA, Laine MK, Huovinen P, Ruuskanen O, Ruohola A. Role of nasopharyngeal bacteria and respiratory viruses in acute symptoms of young children. *Pediatr Infect Dis J*. 34(10), 1056-1062 (2015).

7. Thomas E, Mattila JM, Lehtinen P, Vuorinen T, Waris M, Heikkinen T. Burden of respiratory syncytial virus infection during the first year of life. *J Infect Dis*. 223(5), 811-817 (2021).

8. Okiro EA, Ngama M, Bett A, Nokes DJ. The incidence and clinical burden of respiratory syncytial virus disease identified through hospital outpatient presentations in Kenyan children. *PLoS One*. 7(12), e52520 (2012).

9. Heikkinen T, Ojala E, Waris M. Clinical and socioeconomic burden of respiratory syncytial virus infection in children. *J Infect Dis*. 215(1), 17-23 (2017).

10. Omer SB, Bednarczyk R, Kazi M *et al*. Assessment and validation of syndromic case definitions for respiratory syncytial virus testing in a low resource population. *Pediatr Infect Dis J*. 38(3), e57-59 (2019).

11. Bashir U, Alam MM, Sadia H, Zaidi SS, Kazi BM. Molecular characterization of circulating respiratory syncytial virus (RSV) genotypes in Gilgit Baltistan Province of Pakistan during 2011-2012 winter season. *PLoS One*. 8(9), e74018 (2013).

12. Sáez-López E, Pechirra P, Costa I *et al*. Performance of surveillance case definitions for respiratory syncytial virus infections through the sentinel influenza surveillance system, Portugal, 2010 to 2018. *Euro Surveill*. 24(45), 1900140 (2019).

13. Zar HJ, Nduru P, Stadler JAM *et al*. Early-life respiratory syncytial virus lower respiratory tract infection in a South African birth cohort: epidemiology and effect on lung health. *Lancet Glob Health*. 8(10), e1316-1325 (2020).

14. Haddadin Z, Rankin DA, Lipworth L *et al*. Clinical characteristics of common respiratory viruses detected in infants across different clinical settings. *Open Forum Infect Dis*. 7(suppl 1), S756-757 (2020).

15. Ng KF, Tan KK, Sam ZH, Ting GS, Gan WY. Epidemiology, clinical characteristics, laboratory findings and severity of respiratory syncytial virus acute lower respiratory infection in Malaysian children, 2008-2013. *J Paediatr Child Health*. 53(4), 399-407 (2017).

16. Niles D, Larsen B, Balaji A *et al*. Retrospective review of clinical and chest x-ray findings in children admitted to a community hospital for respiratory syncytial virus infection. *Clin Pediatr (Phila)*. 57(14), 1686-1692 (2018).

17. Bamberger E, Srugo I, Abu Raya B *et al*. What is the clinical relevance of respiratory syncytial virus bronchiolitis?: findings from a multi-center, prospective study. *Eur J Clin Microbiol Infect Dis*. 31(12), 3323-3330 (2012).

18. Pale M, Nacoto A, Tivane A *et al*. Respiratory syncytial and influenza viruses in children under 2 years old with severe acute respiratory infection (SARI) in Maputo, 2015. *PLoS One*. 12(11), e0186735 (2017).

19. Van Leeuwen JC, Goossens LK, Hendrix RM, Van Der Palen J, Lusthusz A, Thio BJ. Equal virulence of rhinovirus and respiratory syncytial virus in infants hospitalized for lower respiratory tract infection. *Pediatr Infect Dis J*. 31(1), 84-86 (2012).

20. Faber TE, Kamps AW, Sjoerdsma MH, Vermeulen S, Veeger NJ, Bont LJ. Computerized assessment of wheezing in children with respiratory syncytial virus bronchiolitis before and after hypertonic saline nebulization. *Respir Care*. 60(9), 1252-1256 (2015).

21. Haddadin Z, Rankin DA, Yanis A *et al*. Respiratory syncytial virus acute respiratory infections in young children in Jordan: a prospective surveillance study. *Open Forum Infect Dis*. 7(suppl 1), S430-431 (2020).

22. Caserta MT, Qiu X, Tesini B *et al*. Development of a global respiratory severity score for respiratory syncytial virus infection in infants. *J Infect Dis*. 215(5), 750-756 (2017).

23. Zhang XB, Liu LJ, Qian LL *et al*. Clinical characteristics and risk factors of severe respiratory syncytial virus-associated acute lower respiratory tract infections in hospitalized infants. *World J Pediatr*. 10(4), 360-364 (2014).

24. Cunningham S, Piedra PA, Martinon-Torres F *et al*. Nebulised ALX-0171 for respiratory syncytial virus lower respiratory tract infection in hospitalised children: a double-blind, randomised, placebo-controlled, phase 2b trial. *Lancet Respir Med*. 9(1), 21-32 (2021).

25. Uusitupa E, Waris M, Heikkinen T. Association of viral load with disease severity in outpatient children with respiratory syncytial virus infection. *J Infect Dis*. 222(2), 298-304 (2020).

26. Bhuiyan MU, Luby SP, Alamgir NI *et al*. Costs of hospitalization with respiratory syncytial virus illness among children aged <5 years and the financial impact on households in Bangladesh, 2010. *J Glob Health*. 7(1), 010412 (2017).

27. Zhou L, Xiao Q, Zhao Y, Huang A, Ren L, Liu E. The impact of viral dynamics on the clinical severity of infants with respiratory syncytial virus bronchiolitis. *J Med Virol*. 87(8), 1276-1284 (2015).

28. Chu HY, Katz J, Tielsch J *et al*. Respiratory syncytial virus infection in infants in rural Nepal. *J Infect*. 73(2), 145-154 (2016).
